# Impact of Technical Sources of Variation on the Hand Microbiome Dynamics of Healthcare Workers

**DOI:** 10.1371/journal.pone.0088999

**Published:** 2014-02-14

**Authors:** Mariana Rosenthal, Allison E. Aiello, Carol Chenoweth, Deborah Goldberg, Elaine Larson, Gregory Gloor, Betsy Foxman

**Affiliations:** 1 Department of Epidemiology, School of Public Health, University of Michigan, Ann Arbor, Michigan, United States of America; 2 Department of Ecology and Evolutionary Biology, University of Michigan, Ann Arbor, Michigan, United States of America; 3 School of Nursing and Department of Epidemiology, Mailman School of Public Health, Columbia University, New York, New York, United States of America; 4 Department of Biochemistry, Schulich School of Medicine and Dentistry, London, Ontario, Canada; University of Massachusetts, United States of America

## Abstract

We assessed the dynamics of hand microbial community structure of 34 healthcare workers from a single surgical intensive care unit over a short (3 week) time period, whilst taking into account the technical sources of variability introduced by specimen collection, DNA extraction, and sequencing. Sample collection took place at 3 different time points. Only the sampling collection method appeared to have a significant impact on the observed hand microbial community structure among the healthcare workers. Analysis of samples collected using glove-juice showed a slightly more similar microbial composition within individual hand samples over time than between the hands of different individuals over time. This was not true for samples collected using a swab, where samples from a single individual were no more similar to each other over time than those among other individuals over time, suggesting they were essentially independent. DNA extraction techniques (lysozyme only versus enzyme cocktail) and sequencing (replicate set 1 versus 2) using Ion Torrent Personal Genome Machine, were not influential to the microbial community structures. Glove-juice sample collection may likely be the method of choice in hand hygiene studies in the healthcare setting.

## Introduction

The human skin is made up of dermal layers, hairs, nerves, glands, and a complex ecosystem of microorganisms, the microbiota. Next-generation sequencing techniques have made characterization of the microbiota rapid and economically feasible, leading to a surge of studies. From these studies, including those funded by the first phase of The Human Microbiome Project (HMP), we are gaining an increasingly complete picture of the skin microbiota. Here, we address the biological variation of the hand microbiota, comparing the dynamics of the individual healthcare worker's (HCW) hand microbiota over time versus among individuals. This is challenging because true biological variation can be obscured by technical variation, for example due to specimen collection technique, DNA extraction methods, and sequencing error. Thus, obtaining an accurate profile of the true, biological hand microbiota dynamics requires an initial assessment of the variation caused by technical sources.

Earlier studies suggest that the composition of hand microbiota varies widely. A study of the hands of 51 healthy, undergraduate students sampled after taking an examination, found an average of 158 unique bacterial phylotypes per hand: only 17% were shared between the two hands of an individual, and 13% between individuals [1]. A high level of intra-personal variability in hand microbiota was also found by Caporaso and colleagues, who compared the right and left palms of two individuals over several months: the phylotypes present on each hand were not significantly correlated (at the species level) [2]. However, the way in which skin samples are collected can impact the diversity of the microbiota. While Grice and colleagues found that over 97% of 16S rRNA sequences obtained from swab, scrape and punch biopsy skin samples were shared, unique operational taxonomic units (OTU) were identified by each sampling technique [3]. DNA is more easily extracted from Gram negative than Gram positive cells [4]. Representation of microbial diversity differed between each of 6 different DNA extraction methods done on 11 human-associated bacterial strains, separately and mixed together [5]. Sequencing, regardless of platform, also introduces errors in terms of obtaining an accurate microbiota profile [6]. The Ion Torrent Personal Genome Machine (PGM) is a relatively new technology with a sequencing error rate comparable to the Roche 454 platforms [7]. However, to date, no metagenomic study of the human microbiome using the PGM has investigated the impact of its error rate on assessments of microbial community structure. Moreover, to our knowledge, no human skin microbiome study has determined the extent to which the true biological variability of the skin microbiota is confounded by these technical sources of variation (sampling collection technique, DNA extraction method, and sequencing).

Understanding the biological variability of the skin microbiome of the hands of HCWs is particularly important for gaining insight into the role of skin microbiota in resisting or enhancing colonization by pathogens [8]. Additionally, the ecological relationship between the hand microbiota, transient contaminants/colonizers, and pathogens, may modify potential for pathogen transmission to other HCWs and/or patients, despite their generally elevated hand hygiene efforts. In this study, we assess the dynamics of hand microbial community structure of 34 HCWs at a surgical intensive care unit over a short (3 week) time period, to determine whether the variability within HCWs over time is less than the difference among HCWs. We address the gap in understanding the impact of potential technical sources of variation in this assessment, by taking into account the variability introduced by specimen collection techniques, DNA extraction methods, and sequencing. Specifically, we compared: 1) a swab versus glove-juice (i.e. the buffer obtained from the sterile bag within a participant's hand had been immersed and massaged) sampling technique, 2) DNA extraction by lysozyme only versus an enzyme cocktail, and 3) sequencing one replicate versus another using Ion Torrent PGM.

## Methods

### Ethics Statement

All participants received detailed information about the study and gave written, informed consent. The study protocol was reviewed and approved by the institutional review board of the University of Michigan (IRBMed #HUM00042622).

### Study Population

Healthcare workers were recruited from the University of Michigan Hospital Surgical Intensive Care Unit (SICU). This is a 20-bed critical care unit that specializes in patient recovery after major post-operative procedures (e.g. transplants, aneurysm repairs, resections, vascular endarterectomies, and amputations) or those requiring extensive physiological monitoring. The SICU also accommodates patients from other surgical units (trauma-burn, neurosurgery, medical, and cardiovascular). To qualify for inclusion, volunteers had to be a HCW in the SICU, and not have received topical or systemic steroids or antibiotics for 3 months before the start of the study. Physicians were excluded from the study due to their high mobility. The study was presented at staff meetings and the first 35 HCWs who met eligibility criteria and gave written consent were included in the study. One HCW was lost to follow-up prior to sample collection leaving a total sample size of 34. The study took place July 5–28, 2011.

### Sample Collection

A total of 3 samples were collected from each of the 34 HCWs at different time points, resulting in a total of 102 samples per collection method (*i.e.*, swab and glove-juice) [Supporting Figure 1]. One negative control consisting only of buffer solution (20 mM Tris pH 92 8, 2 mM EDTA, and 1.2% Triton X-100) was produced for each time point, resulting in a total of 3 negative controls per collection method. Sample collection took place at the SICU, where HCWs were randomly sampled at the start, middle, and end of their 12-hour work shifts. To minimize sample cross-contamination the study recruiters donned a new pair of sterile gloves prior to each sample collection. The palm, fingertip surfaces, and in-between the fingers of the participant's dominant hand were swabbed using sterile cotton-tipped swabs soaked in the buffer solution. Swabbing was performed in two perpendicular directions to ensure that the maximum surface area was represented in the sample. Immediately after swabbing, the participant's dominant hand was inserted into a sterile, polyethylene bag containing 50 ml buffer solution (0.07 M PBS, 0.1% Tween-80) and massaged through the wall of the bag for 1 minute. The buffer solution, here termed glove-juice, was then collected. All samples were stored at −20°C until further processing.

### DNA Extraction, Purification and Amplification

All controls, swab samples, and the pellet of 1 ml of all glove-juice samples were lysed using enzyme cocktail (mutanolysin @ 160 U/ml, Rnase A @ 0.07 mg/ml, lysostaphin @ 0.16 mg/ml, and lysozyme @ 7 mg/ml) for 30 minutes at 37°C. A control and a subset of ten glove-juice samples from the first collection were lysed per manufacturer's recommendations using only lysozyme @ 20 mg/mL for 30 minutes at 37°C. The standard protocol for lysing gram-positive bacterial cell lysates of the PureLink Genomic DNA kit (Invitrogen Corp.; #K1820-02) was followed for all subsequent steps, with an additional incubation at 95°C for 2 minutes, prior to the addition of 96–100% ethanol to the lysates. This protocol, though not one used by the HMP, was chosen due to previous successful bacterial extraction and purification from skin surface [9]. Purified genomic DNA were re-suspended in 50 µl of PureLink Genomic Elution Buffer and stored at −80°C until sent for sequencing.

DNA was tested for PCR competency using the following procedure. The primers L-V6 (5′-CAACGCGARGAACCTTACC-3′) and R-V6 (5′-CAACACGAGCTGACGAC-3′) were chosen to amplify the V6 hypervariable region of the 16S rRNA gene [10]. After extraction, 1 uL of the purified genomic DNA was used as template for a 25 uL PCR reaction on a MyCycler Thermal Cycler (Bio-Rad Laboratories, Inc.). The following PCR reactions were used: 22.5 ul of Platinum Blue PCR SuperMix (Invitrogen Corp., #12580-023) 1 ul of 10 uM primer pair, and 0.5 ul of water. PCR conditions included: 94°C for 2 minutes; 30 cycles of [94°C for 30 seconds; 55°C for 30 seconds; 72°C for 30 seconds]; and hold at 4°C. A negative control including all ingredients but with water instead of DNA template was included alongside all test reactions. A constant volume aliquot of each PCR amplification product was run on a 1.5% agarose gel to determine PCR competency as well as the approximate amount of product. 10–20 ul of the purified genomic DNA were sent for sequencing at The London Regional Genomics Centre at the University of Western Ontario (London, ON, Canada).

### DNA Preparation for Sequencing

The bacterial V6 rRNA region was amplified with the left-side primer CWACGCGARGAACCTTACC and the right-side primer ACRACACGAGCTGACGAC. These primer sequences are exact matches to >95% of the rRNA sequences from organisms identified in the human microbiome project. The left-side primers contained the standard Ion Torrent (Ion Torrent Systems, Guilford, CT, USA) adapter and key sequence at their 5′ end (CCATCTCATCCCTGCGTGTCTCCGACTCAG). The right-side primer had the other standard Ion Torrent adapter sequence (CCTCTCTATGGGCAGTCGGTGAT) attached to its 5′ end. Amplification was performed for 25 cycles in 40 µl using the colorless GO-Taq hot start master mix (Promega; #M5133) according to the manufacturer's instructions with the following three-step temperature profile: 95°C, 55°C and 72°C for 1 minute each step. 5 µl of the resulting amplification were quantified using the QuBit broad-range double-stranded DNA fluorometric quantitation reagent (Invitrogen Corp.; #Q32854). Samples were pooled at approximately equal concentrations and purified using a Wizard PCR Clean-Up Kit (Promega; #A9285).

### DNA Sequencing and Sequence Reads Filtering

Sequencing reactions were carried out on three Ion Torrent 316 platform chips, multiplexing up to 96 samples per run using the 200 bp sequencing reagent kit. Data from all runs were pooled. The sequence was provided in fastq format. All sequences were filtered according to the following criteria in order: exact match to the left-side primer including redundant positions in the primer, exact matches to the barcodes used, an exact match to the first six nucleotides of the right-side primer, and a length between the left-side and right-side primer of between 71 and 90 nucleotides. This length was chosen because it encompasses the predicted amplicon product size from all human-associated bacterial organisms that have been cultured and sequenced as part of the HMP. Table 1 shows the number of raw and filtered reads obtained from each run. Run number 3 had the least number of sequences because of sub-optimal loading efficiency. However, as the reproducibility of the Ion Torrent platform for these types of analyses is excellent provided the number of reads per sample is greater than 1000 [11], this was not a concern.

**Table 1:** Number of Raw and Processed Sequencing Reads per Ion Torrent Personal Genome Machine (PGM) Sequencing Run, Using 316 Chips, of 280 Samples of Hand Microbiota from 34 Healthcare Workers at the University of Michigan Surgical Intensive Care Unit, July 5–28, 2011.

Sequencing Run	Raw Sequence Reads	Processed Sequence Reads	Proportion of Processed/Raw Sequence Reads
1	2,787,276	1,292,855	0.464
2	3,160,031	2,132,925	0.675
3	903,240	643,015	0.712

Between 46 to 71% of the reads passed these filters; reads not passing the filters were not examined further. Reads were processed as previously described [12] except that clustering with USEARCH was performed at 97% identity. Chimera detection was performed with UCHIME (version v5.2.32) using the de novo method [13]. Chimeric sequences in less than 0.05% in any sample were discarded. A table of counts for sequences grouped at 100% identical sequence unit (ISU) identity level were generated for each sample [12], keeping all sequences that were represented in any sample at a frequency >0.5%. Reads that were never abundant in any sample (<0.5%) were discarded. Data for this experiment have been deposited in the European Nucleotide Archive under accession number PRJEB5147.

### Taxonomic Classification

Classification of the sequences by either the Greengenes or RDP Bayesian classifiers proved to be unreliable because of the short length of the V6 region. Classification of the representative OTU sequences present in the count table was therefore performed using the kmer-based RDP Seqmatch tool [14] using the following options: both type and non-type strains; isolates only; length greater than 1200; good quality; nomenclatural taxonomy. The 20 best KNN (kmer nearest neighbor) hits were identified, and the taxonomic classification of the best match and ties was noted. The classification of the best hit and ties for the OTU sequence was adopted for all taxonomic levels where the classification was identical across all the best-hit KNN matches. For example, if the best KNN hits and ties were identical to the genus level, but differed at the species level then the OTU was annotated to the genus level and the species was labeled as undefined. The taxonomic classification was added to the sequence count table and the data imported into QIIME 1.5.0 [15]. Sequence alignments were built using Muscle [16] and a neighbor-joining tree was generated by ClustalW2 [17].

### Statistics

Quantitative Insights into Microbial Ecology (QIIME, version 1.5.0), an open source software package for comparison and analysis of microbial communities, was used to process data from the Ion Torrent sequence reads. Analyses included removal of chloroplast sequences to the development of taxonomic summaries of communities, computing of alpha diversities, rarefaction curves, principal coordinate analyses (PCoA), distance histograms, jackknifed bootstrapping of beta diversities, and analysis of similarities (ANOSIM). Rarefied operational taxonomic unit (OTU) tables were generated to compute measures of alpha diversity. Metrics computed were Chao1, which estimates the species richness; observed species, which counts the number of unique OTUs in a sample; Shannon index, which estimates the species diversity; and PD_whole_tree, a phylogenetic distance metric. Rarefaction curves, showing the alpha diversity versus simulated sequencing effort, were generated.

To compare the bacterial communities between groups, beta diversity metrics were calculated based on the UniFrac algorithm, which measures the community similarity based on shared branch length on a phylogenetic tree [18]. To remove sample heterogeneity and standardize comparisons so that sequencing effort does not influence diversity estimates, the OTU tables were rarefied. Weighted UniFrac dissimilarity matrices of each comparison group formed the basis for the distance histograms, distance boxplots, and PCoA's. The distribution of weighted UniFrac distances within one group was displayed in a histogram, and overlaid with the distribution of distances between groups. Boxplots comparing distances within and between groups were generated from the sets of weighted UniFrac distance matrices. Jackknife bootstrapping was performed to estimate the uncertainty in the PCoA plots. Two statistical approaches were used to compare phylogenetic composition based on the UniFrac distance matrices between groups. We first conducted an ANOSIM, which is a modified version of the Mantel Test based on a standardized rank of correlation between two distance matrices [19]. Second, we took into account the hierarchical sampling design by conducting a paired t-test using the first principal components of the PCoA plots. The pairs assessed differences due to one of the technical sources of variation, but within a particular sample taken from a given HCW at a given time.

## Results

**Figure 1:**
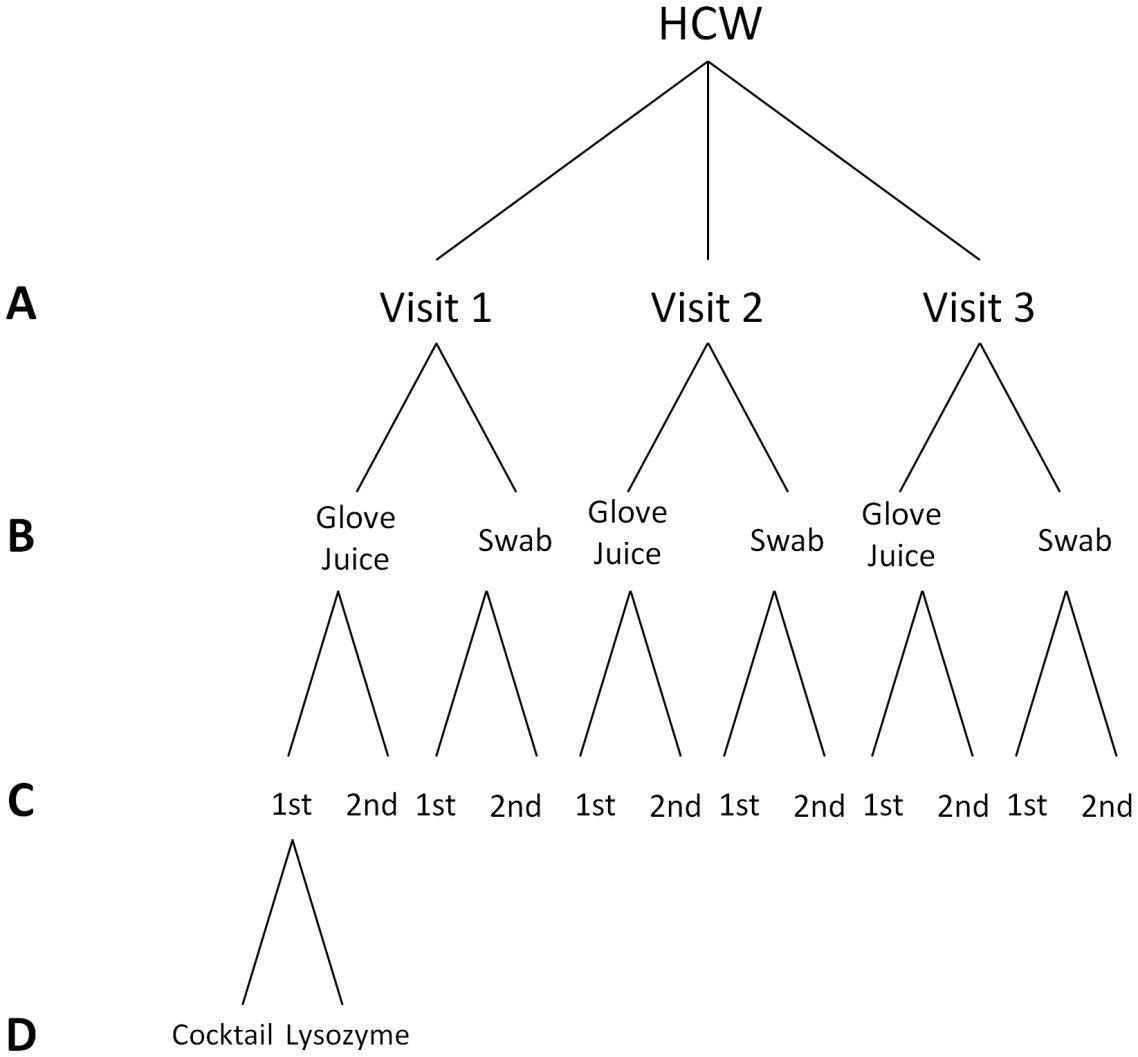
Study Design Showing Levels of Comparisons of Hand Microbiota Samples Sent for Sequencing. Level A shows the comparison of within versus between HCWs (n_1_ = 34, n_2_ = 34, n_3_ = 34); level B shows the comparison of sampling collection methods (n_SW_ = 102, n_GJ_ = 102); level C shows the comparison of sequencing replicates (n_1_ = 30, n_2_ = 30); and, level D shows the comparison of DNA extraction methods (n_C_ = 10, n_L_ = 10). Samples obtained from 34 Healthcare Workers at the University of Michigan Surgical Intensive Care Unit, July 5–28, 2011.

We assessed the dynamics of hand microbial community structure of 34 HCWs over a 3 week period while considering the variability introduced by sampling collection method, DNA extraction method, and sequencing [Figure 1]. During analysis, DNA sequence identity level was kept at 100% so that true differences between microbial communities could be assessed in the several comparisons that follow. Moreover, all comparisons were made within the same OTU dataset without stratification, so as to control for the variability observed elsewhere. The mean number of sequencing reads assigned to the OTU table was 6,514 per sample (min = 4, max = 77,185).

### Comparison of Sampling Collection Method

**Figure 2:**
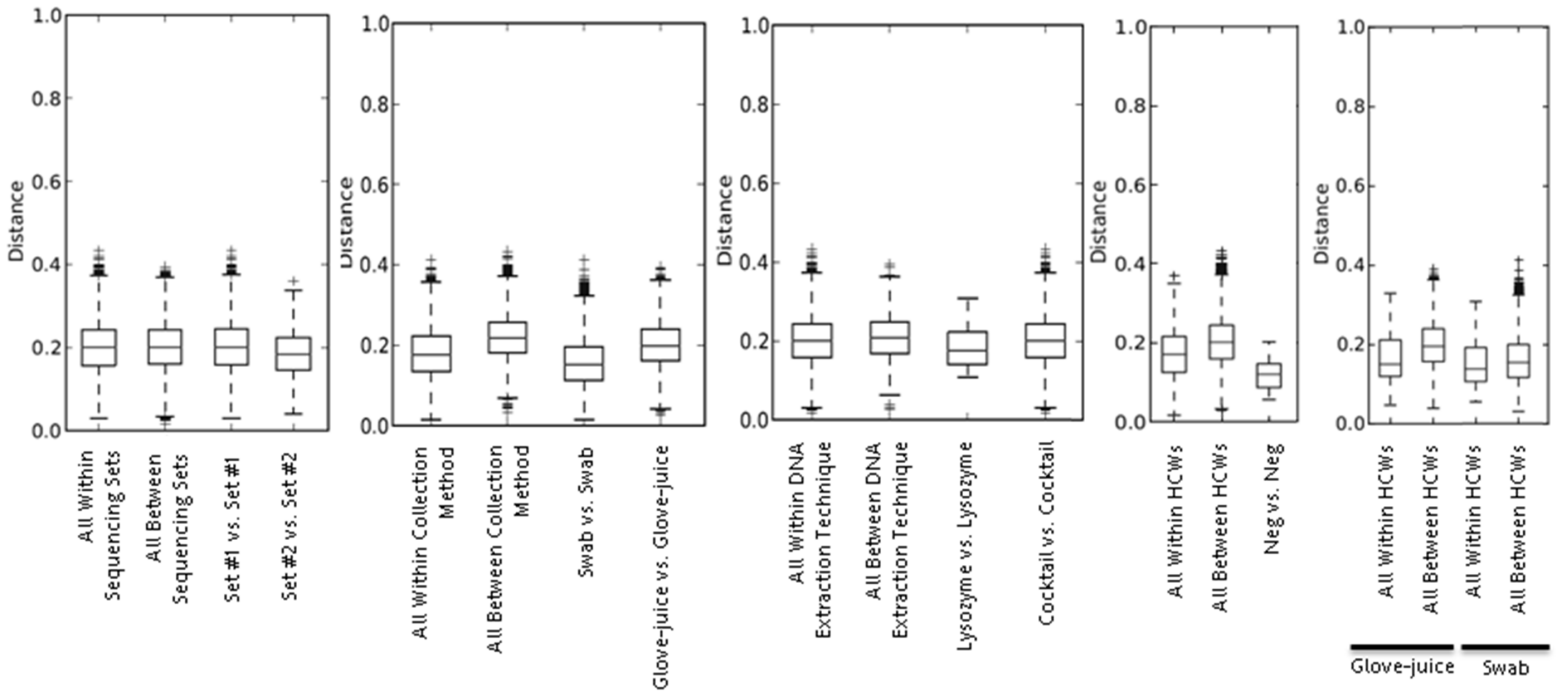
Within and Between Weighted UniFrac Distances of the Hand Microbiota. Stratification by Sampling Collection Method (Panel A: Glove-Juice and Swab), DNA Extraction Method (Panel B: Lysozyme and Cocktail), Sequencing Replicates (Panel C: Set #1 and Set #2), Healthcare Workers (Panel D: Within and Between), and Healthcare Workers by Sampling Collection Method (Panel E: Within and Between). Samples obtained from 34 Healthcare Workers at the University of Michigan Surgical Intensive Care Unit, July 5–28, 2011.

**Figure 3:**
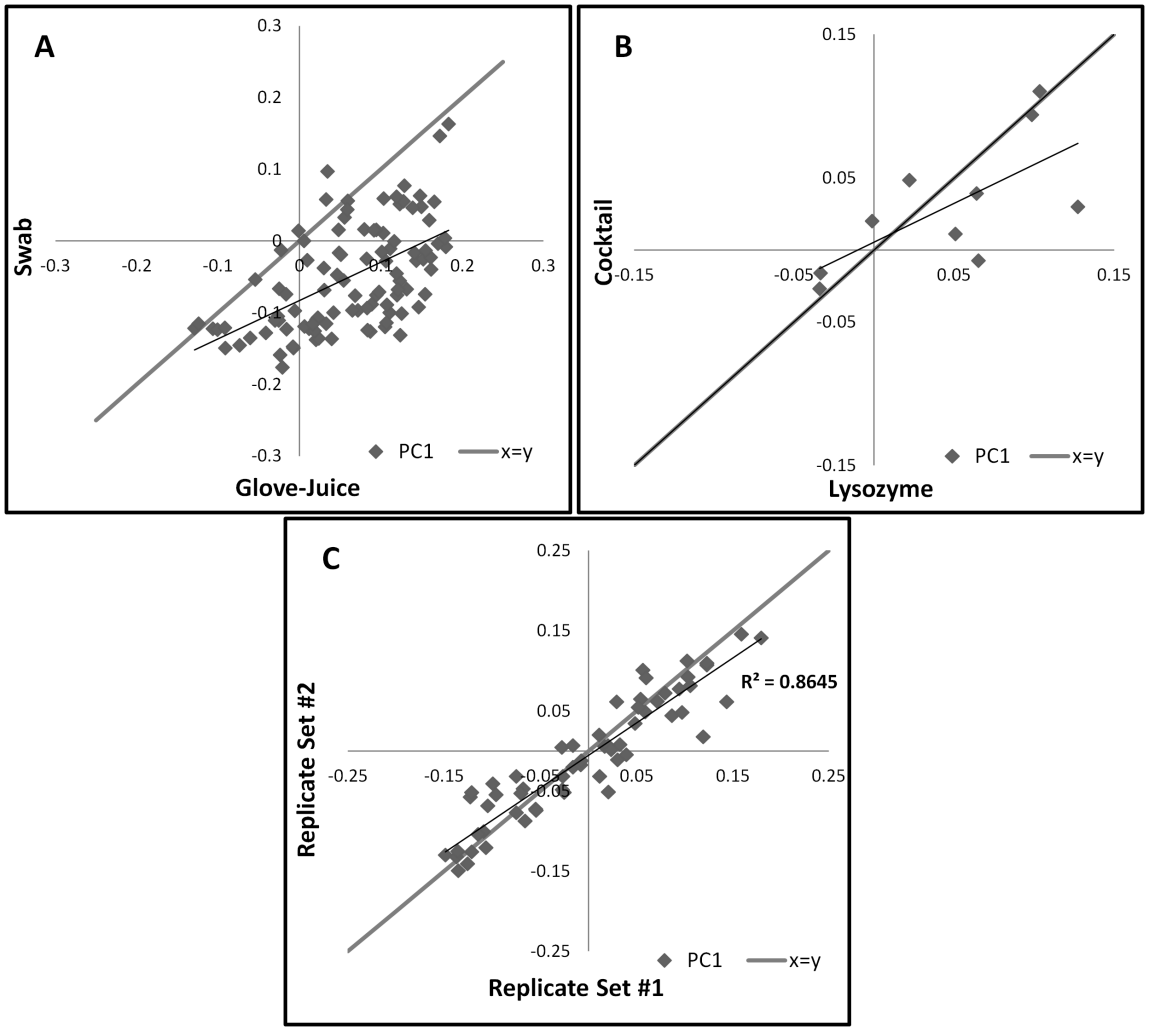
First Principal Components Scatterplots of the Principal Coordinate Analysis (weighted UniFrac) of the Hand Microbiota. Stratification by Sampling Collection Method (Panel A: Glove-Juice (x-axis) and Swab (y-axis)), DNA Extraction Method (Panel B: Lysozyme (x-axis) and Cocktail (y-axis)), and Sequencing Replicates (Panel C: Set #1 (x-axis) and Set #2 (y-axis)). Samples obtained from 34 Healthcare Workers at the University of Michigan Surgical Intensive Care Unit, July 5–28, 2011.

At each visit, samples were first collected via swabs and immediately after, via glove-juice, totaling 102 samples per collection method. Comparisons of alpha diversity suggested that the differences between the two methods were small [Supporting Figures 2 and 3]. The total average number of unique phylotypes obtained by glove-juice and swab was 129 and 125, respectively (t = 1.32, p = 0.19). Further analyses, however, revealed some important differences [Supporting Figures 4–7]. Boxplots indicate that the mean weighted UniFrac distance between the two sampling collection methods is higher than the mean weighted UniFrac distance within either of the two methods, indicating a meaningful difference between them [Figure 2], and ANOSIM results show a statistically significant difference between the weighted UniFrac distance matrices (R = −0.2649, p<0.001) [Table 2]. A scatterplot of the first principal component of the PCoA comparing both sampling collection methods from an individual HCW at a given time show most coordinates falling to the right of the expected line (y = x), indicating that the two sets are not equivalent (paired t = 10.51, p<0.001) [Figure 3].

**Table 2:** ANOSIM of the Hand Microbiota from 34 Healthcare Workers at the University of Michigan Surgical Intensive Care Unit, July 5–28, 2011, Comparing Sampling Collection Method (Glove-Juice and Swab), DNA Extraction Method (Lysozyme and Cocktail), and Sequencing Replicates (Set #1 and Set #2).

Group 1	Group 2	R Statistic	p-value
Glove-Juice	Swab	0.2649	<0.001
Lysozyme	Cocktail	0.0901	0.067
Replicate Set #1	Replicate Set #2	0.0122	0.326

### Comparison of DNA Extraction Technique

To test whether DNA extraction techniques influence microbial community structure, the DNA of the first 10 glove-juice samples from the first visit was extracted using two slightly different methods. One method used lysozyme (20 mg/ml) only; the other, an enzyme cocktail comprising of mutanolysin (60 U/ml), Rnase A (0.07 mg/ml), lysostaphin (0.16 mg/ml), and lysozyme (7 mg/ml). While boxplots of the mean weighted UniFrac distance between techniques indicate a slight difference between them [Figure 2], PCoA fail to show clear clusters per DNA extraction technique [Supporting Figures 6 and 7]. Moreover, ANOSIM results show no statistically significant difference between the DNA extraction technique sets of weighted UniFrac distance matrices (R = 0.0901, p = 0.067) [Table 2]. Even the paired analysis, comparing the first principal component of the PCoA from a single sample between the two DNA extraction techniques show most coordinates falling around the expected line (y = x), also indicating that the two sets are equivalent (paired t = −0.68, p = 0.5047) [Figure 3].

### Comparison of Sequencing Replicates

Duplicate sets of the first 10 samples from each visit (n = 30) were sent for sequencing. Sequencing replicates had similar relative abundances of taxa, and equivalent average alpha diversity, indicating consistent sequencing results [Supporting Figures 2 and 3]. Other tests, including a histogram comparing weighted UniFrac distances, and PCoA plots performed with jackknife bootstrapping, suggested no differences between the replicates [Supporting Figures 4–7]. Boxplots of the mean weighted UniFrac distance indicate no difference between the sequencing replicates [Figure 2] and ANOSIM results show no statistically significant difference between the sets of weighted UniFrac distance matrices (R = 0.0122, p = 0.326) [Table 2]. A scatterplot of the first principal component of the PCoA comparing both replicate sets show most coordinates falling around the expected line (y = x), indicating that the two sets are equivalent (paired t = 0.36, p = 0.7536) [Figure 3].

### Comparison of between versus within Healthcare Worker

To assess the biological variability of hand microbial community structure within and between HCWs, we sampled participants at 3 time points. Since significant differences were observed between samples collected via glove-juice and swab, within versus between HCW comparisons were stratified by sampling collection method. The difference in mean weighted UniFrac distances within and between HCWs by sampling collection method, as shown by the boxplots of weighted UniFrac distances, is more pronounced among the glove-juice samples, where the mean weighted UniFrac distance between HCWs is much higher than within HCWs [Figure 2]. A two-sample t test comparing weighted UniFrac distances within versus between HCWs, found a significant difference among the samples collected via glove-juice (t = 5.35, p-value <0.0001) but not swabs (t = 1.43, p-value = 0.1516).

## Discussion

Analysis of the microbiome of 34 HCWs tested weekly over 3 weeks showed variability between and within HCWs that could not be attributed to technical variation introduced by sampling collection method, DNA extraction technique, or sequencing. Ours is the first study, to our knowledge, that has compared overall microbial composition between two different hand sampling methods that have been used in the hand hygiene literature for identifying bacterial counts and pathogens on the hands. The observed variability in microbial community structure based on sampling method has important implications for the interpretation and future methodology of microbial composition studies in both hand hygiene and microbiome literature. We also showed that the two DNA extraction techniques resulted in slightly different beta diversity profiles of the hand microbiome, albeit not statistically significant, possibly due to a low sample size. Last, our sequencing results indicated that duplicate samples sequenced in different runs using the Ion Torrent PGM technology were not statistically different, suggesting that this platform is well suited for human metagenomic studies.

In regard to methodology of gathering hand samples, we found that when using swab samples, HCWs' hands appeared as similar in microbial composition to themselves over time as they were to the hands of other HCWs in the study. This is consistent with the study of Caporaso and colleagues, who tested swab samples from both the right and left hands of two individuals over 396 time points and found high variability within an individual across time, and, no significant correlation between the species-level taxa presence on the right palm compared to the left [2]. In contrast, based on samples from glove-juice, the microbiota was slightly more similar within HCWs over time than between HCWs. The increased similarity between glove-juice samples within a HCW may reflect the larger surface area surveyed providing more opportunities for differences between individuals to arise.

It is hospital infection control policy for HCWs to perform hand hygiene upon leaving a patient's room. Although each HCW cared for, on average, one to two patients, and were thus likely exposed to different microbes, it may be that their high level of handwashing and use of alcohol gel were sufficient to remove from their palmar surfaces whatever would differentiate one HCW from another in terms of the microbiota gathered from their patients.

**Figure 4:**
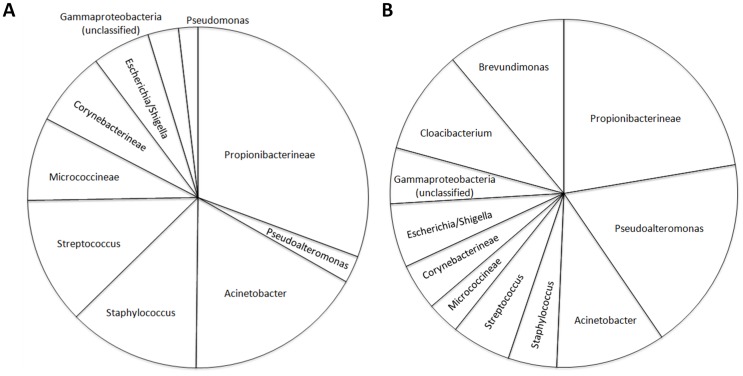
Relative Abundances of the Top 80% Most Abundant Taxa Detected per Sampling Method. Sampling methods (Panel A: Glove-Juice; Panel B: Swab) of the Hand Microbiota are obtained from 34 Healthcare Workers at the University of Michigan Surgical Intensive Care Unit, July 5–28, 2011.

Which sample collection method is preferred depends on the research question. If transmission is presumed to arise solely from direct contact, swabbing may provide adequate representation of the microbiota present. However, if transmission is thought to arise both from direct contact and from shedding of skin cells, then sampling via glove-juice would give a more complete picture of the potential for transmitting both transient and colonizing microbiota. The incidence of infections acquired by patients in an intensive care unit (ICU) is a great public health concern. A 2007 study of the prevalence of infection in 1265 ICUs from 75 countries found that in patients with positive isolates, the most common organisms were *Staphylococcus aureus* (20.5%) and *Pseudomonas* spp. (19.9%) [20]. In our study, we detected a higher abundance of these two bacteria using glove-juice compared to swabs [Figure 4]. On the other hand, overall, there was a positive correlation between the microbial community structure observed from the two sampling methods [Figure 3].

Further work is needed to establish whether the microbiota detected by swabs are indeed nested within the microbiota detected by glove-juice. Of note, prior skin microbiome studies of the Human Microbiome Project (HMP) have mostly used swabs to characterize the microbial communities of the skin. In contrast, hand hygiene studies in healthcare settings generally use the glove-juice method, mostly for obtaining microbial loads for culturing. It is termed the 'gold standard' for infection control as it provides a thorough collection of transient microbial contamination as well as whole hand and nail microbiota [21]. More comparisons of the two sampling collection methods – research that is lacking in the literature – and the dynamics observed in each, would be meaningful for bridging the two research fields.

Our results comparing DNA extraction methods were ambiguous. While we observed trends in composition, these differences were not statistically significant, probably due to the small sample size (n = 10) used in the comparison. DNA extraction can impact the true representativeness of the metagenomic study and the generalizability of results between studies [22]. A recent study using six different DNA extraction methods to compare the profiles of 11 bacterial species and a mock community comprised of all these species found that none were accurate in describing the composition of the mock community [5]. However, they determined that protocols using bead beating and mutanolysin (25 KU/ml) together, best represented the true microbial community structure. We used a lower concentration of mutanolysin (160 U/ml) in our enzyme cocktail, however the cocktail also contained Rnase A, lysostaphin, and lysozyme. Since no mock community was used, we cannot report on the accuracy of using the enzyme cocktail in obtaining the true representativeness of the hand microbiota.

With respect to the assessment of whether the Ion Torrent PGM sequencing platform used introduced variation in the hand microbial community structures within and between HCWs, we found that we obtained the same results for samples sequenced in duplicate. This is a relatively new technology that has not been extensively implemented in microbiome studies. To our knowledge, despite the existence of several papers describing this new platform's performance [23]–[26], only two other metagenomic studies of the human microbiome have been published to date using this platform [27]–[28]. This study is the first skin microbiome study to compare microbiome samples to themselves in order to assess technical variability introduced by the Ion Torrent PGM.

One limitation of our study may be that our sample size may not be sufficient to accurately determine the short-term stability of hand microbiota. Additional samples comparing DNA extraction techniques would also have proven beneficial. However, we argue that any effect that exists is likely to be small, given that we were able to account for known sources of technical variability (e.g. sampling collection, DNA extraction technique, and sequencing). In addition, it would have been preferable to have had the HCWs perform the same hand hygiene protocol before sampling. However, the high frequency of overall hand hygiene per work shift reported among the participants suggests that keeping their handwashing and alcohol rub use constant would not have changed our conclusions. A final limitation is, by necessity, hands were swabbed first followed by sampling using glove-juice. It is possible that swabbing removed the outer layer of skin-associated bacteria that might be recovered using glove-juice sampling. We believe that the impact of this, if any, are small, as the rarefaction of curves of alpha diversities by collection method, were not statistically significant (Supporting Figure 3).

In conclusion, analyses of the microbiota found on HCWs' hands indicate that the observed dynamics of the microbial community structure depends on sample collection method. Using the glove-juice method, hands from within an individual were slightly more similar in microbial composition over time than between individuals. Using swab, samples from a single individual were no more similar to each other than those between individuals. Other sources of technical variation assessed, specifically DNA extraction techniques and sequencing, did not influence the microbial community structures. Future studies of the hand microbiota should consider sampling methods during study design to fit their research question (e.g., expected transmission route). Glove-juice sample collection may likely be the method of choice in hand hygiene studies in the healthcare setting, as it was able to capture higher amounts of known hospital pathogens, and perhaps, a collection of both transients and endogenous bacteria.

## Supporting Information

Figure S1 **Organization of the 280 Hand Microbiota Samples Sent for Sequencing.** Samples obtained from 34 Healthcare Workers at the University of Michigan Surgical Intensive Care Unit, July 5–28, 2011.
(TIF)

Figure S2 **Rarefactions of Phylogenetic Distance (PD_whole_tree) between the Comparison Groups.** Rarefaction curves of phylogenetic distance show that the average alpha diversity is equivalent for both sets of sequencing replicates, and slightly yet not significantly different by collection method and DNA extraction technique.
(TIF)

Figure S3 **Rarefaction Curves of Alpha Diversities per Collection Method.** Measures of average species richness and number of observed species appear higher for samples collected via the glove-juice method, while the average species diversity seemed equal regardless of collection method.
(TIF)

Figure S4 **Average Relative Phylum Abundance per Comparison Groups.** Sequencing replicates #1 and #2 comprised Proteobacteria (46.7%; 40.4%), Actinobacteria (28.9%; 30.5%), Firmicutes (19.7%; 22.4%), and Bacteroidetes (4.7%; 6.1%), respectively. Glove-juice and swab samples comprised Proteobacteria (35.9%; 56.2%), Actinobacteria (38.8%; 24.6%), Firmicutes (23.8%; 11.0%), and Bacteroidetes (1.4%; 8.0%), respectively. Enzyme cocktail and lysozyme-only samples comprised Proteobacteria (38.2%; 25.5%), Firmicutes (30.6%; 37.6%), Actinobacteria (29.7%; 31.9%), and Bacteroidetes (1.5%; 4.5%), respectively.
(TIF)

Figure S5 **Distribution of Weighted UniFrac Distances Between and Within Each Comparison Group.** Weighted UniFrac distance histograms show distribution of distances within sequencing replicate sets similar to the distribution of distances between them. The distribution of distances within sampling collection method was shifted from the distribution of distances between them. The distribution of distances within DNA extraction method was slightly different than the distribution of distances between them.
(TIF)

Figure S6 **2D and 3D Principal Coordinate Analysis (weighted UniFrac) Stratified by Comparison Group.** 2D and 3D PCoA do not show clear clusters per DNA extraction technique (B: Lysozyme (blue) and Cocktail (red)) nor per sequencing replicate set (C: Set #1 (blue) and Set #2 (red)). However, they indicate relative clustering by sampling collection method (A: Glove-Juice (red) and Swab (blue)).
(TIF)

Figure S7 **Jackknifed Principal Coordinate Analysis (weighted UniFrac) per Replicate, Sampling Collection Method, and DNA Extraction Method.** PCoA performed with jackknife bootstrapping shows considerable overlapping of both sequencing replicate sets as well as DNA extraction methods, but relative clustering by sampling collection method.
(TIF)
