# Supplementary figures and images for: Impact of Technical Sources of Variation on the Hand Microbiome Dynamics of Healthcare Workers
Source: PLoS One. 2014 Feb 14;9(2):e88999. doi: 10.1371/journal.pone.0088999 (PMC3925205; doi:10.1371/journal.pone.0088999)

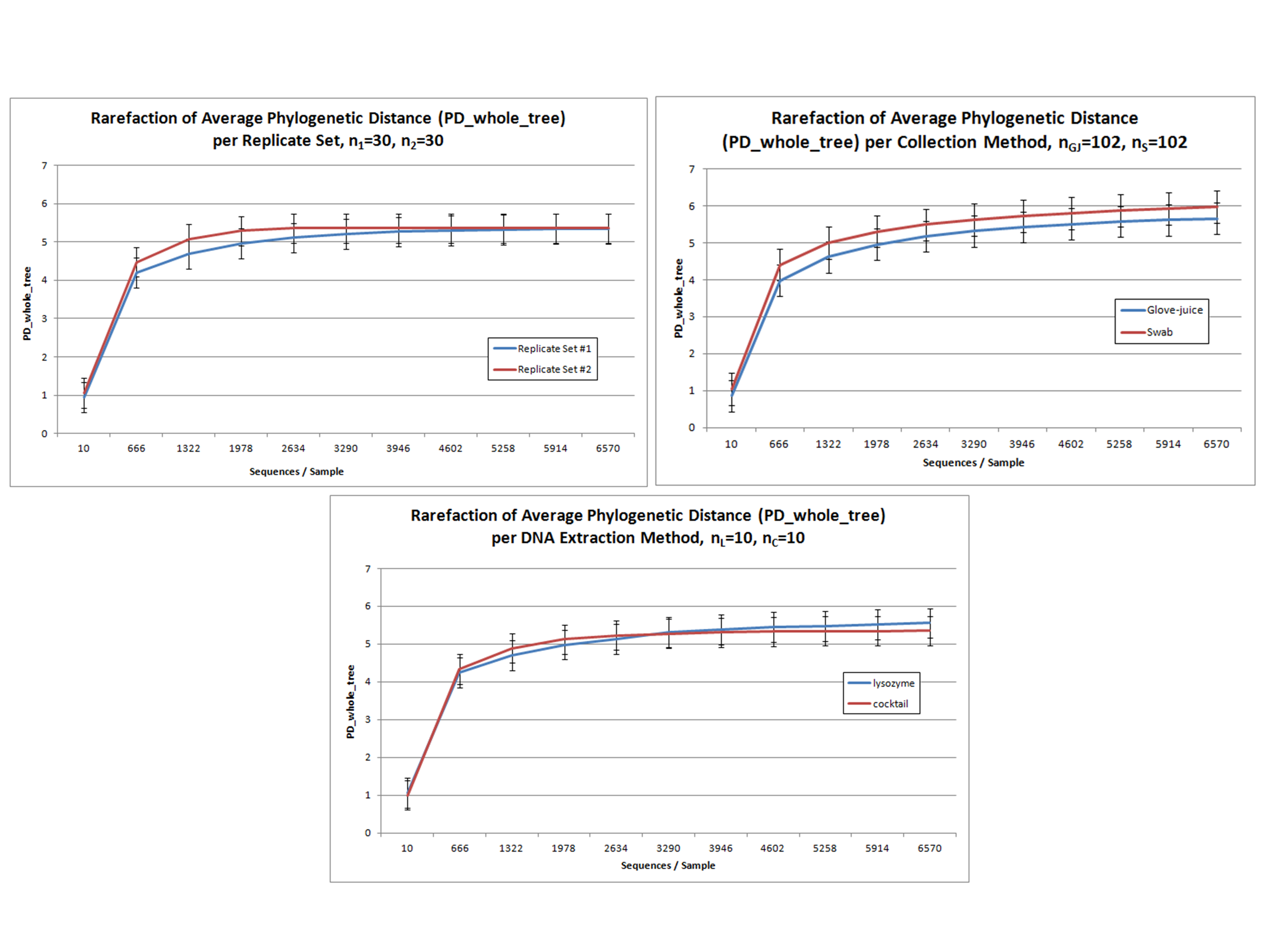

Supplement: Figure S2 — Rarefactions of Phylogenetic Distance (PD_whole_tree) between the Comparison Groups. Rarefaction curves of phylogenetic distance show that the average alpha diversity is equivalent for both sets of sequencing replicates, and slightly yet not significantly different by collection method and DNA extraction technique. (TIF) [file pone.0088999.s002.tif]

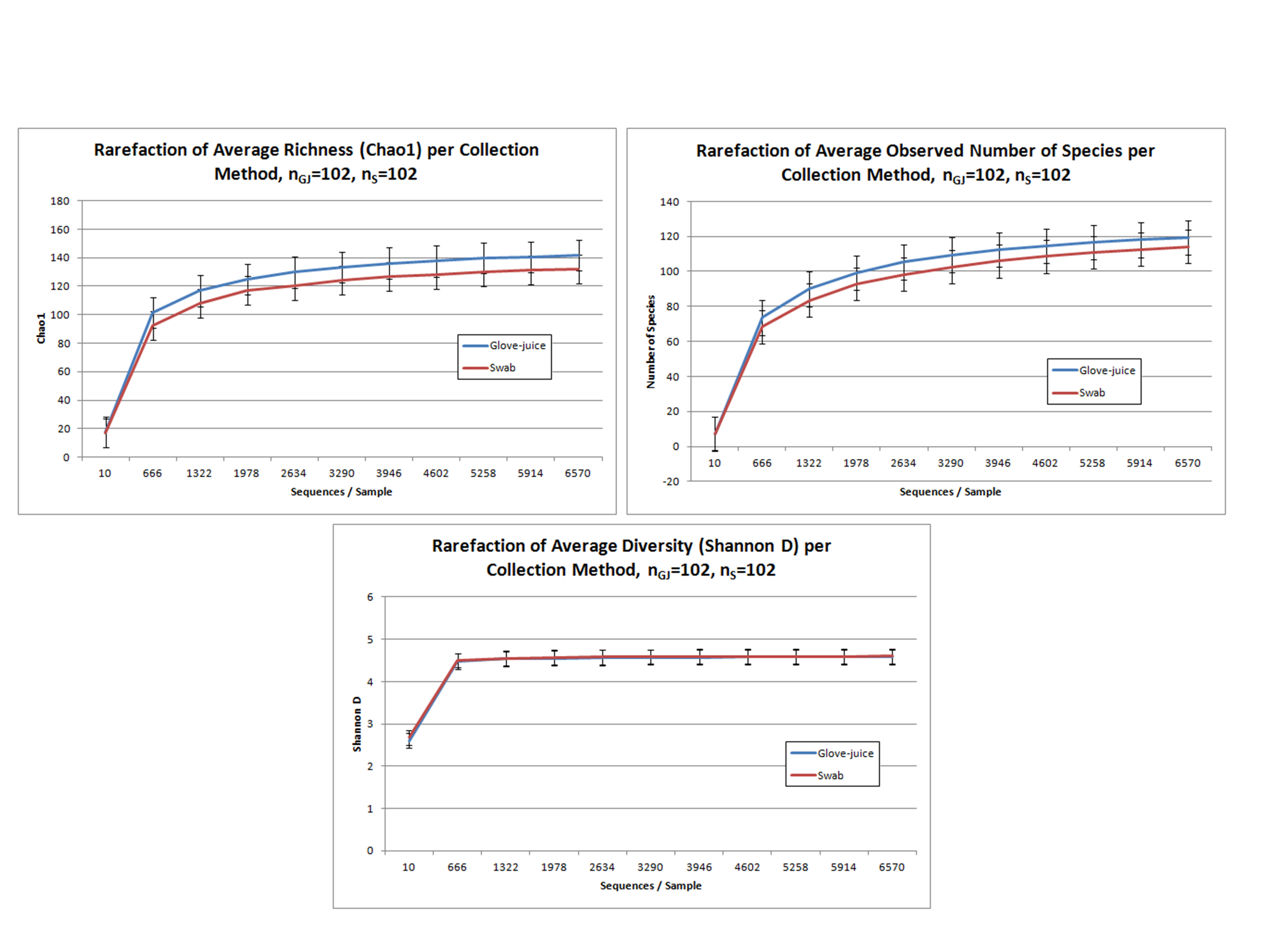

Supplement: Figure S3 — Rarefaction Curves of Alpha Diversities per Collection Method. Measures of average species richness and number of observed species appear higher for samples collected via the glove-juice method, while the average species diversity seemed equal regardless of collection method. (TIF) [file pone.0088999.s003.tif]

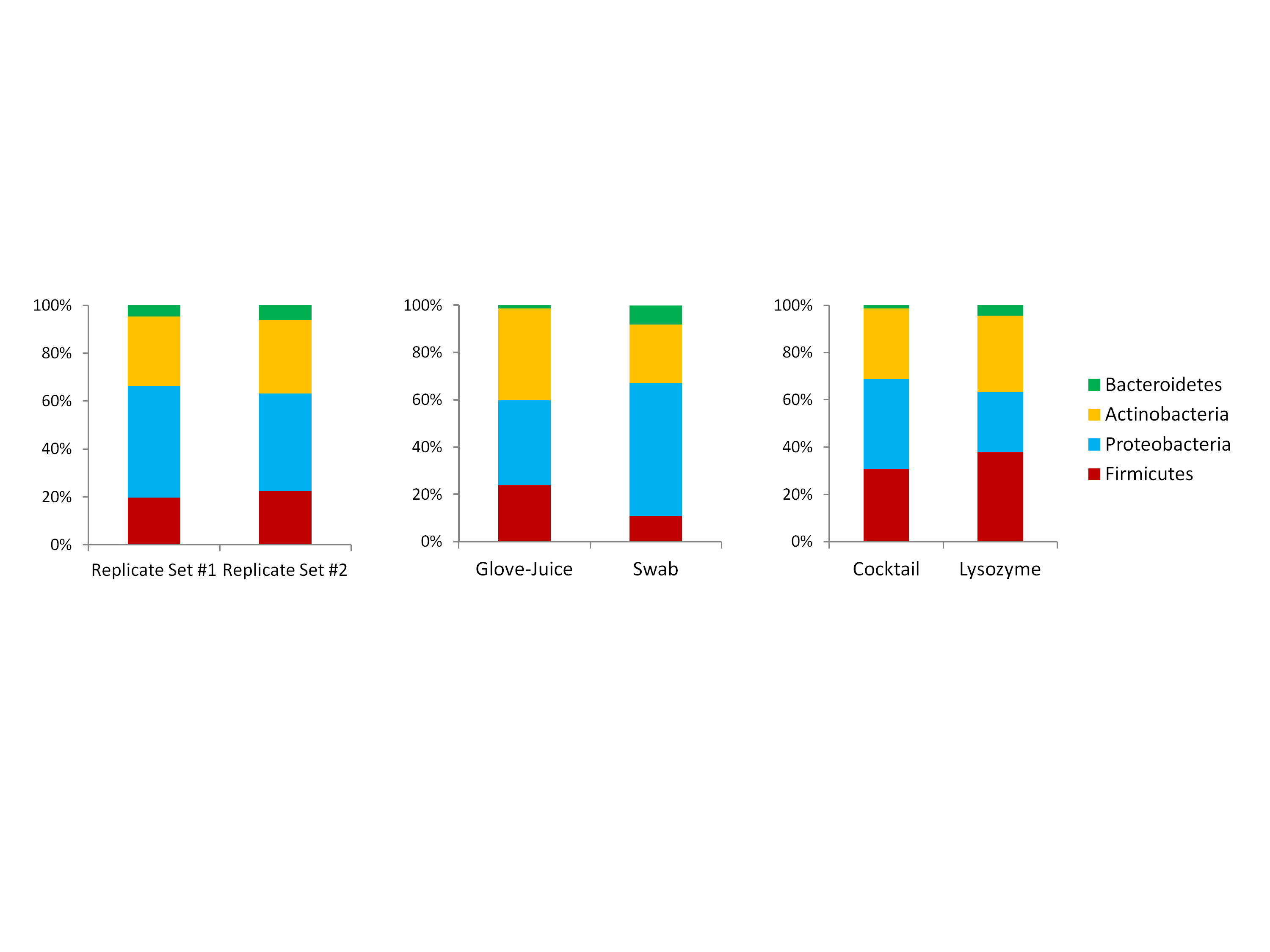

Supplement: Figure S4 — Average Relative Phylum Abundance per Comparison Groups. Sequencing replicates #1 and #2 comprised Proteobacteria (46.7%; 40.4%), Actinobacteria (28.9%; 30.5%), Firmicutes (19.7%; 22.4%), and Bacteroidetes (4.7%; 6.1%), respectively. Glove-juice and swab samples comprised Proteobacteria (35.9%; 56.2%), Actinobacteria (38.8%; 24.6%), Firmicutes (23.8%; 11.0%), and Bacteroidetes (1.4%; 8.0%), respectively. Enzyme cocktail and lysozyme-only samples comprised Proteobacteria (38.2%; 25.5%), Firmicutes (30.6%; 37.6%), Actinobacteria (29.7%; 31.9%), and Bacteroidetes (1.5%; 4.5%), respectively. (TIF) [file pone.0088999.s004.tif]

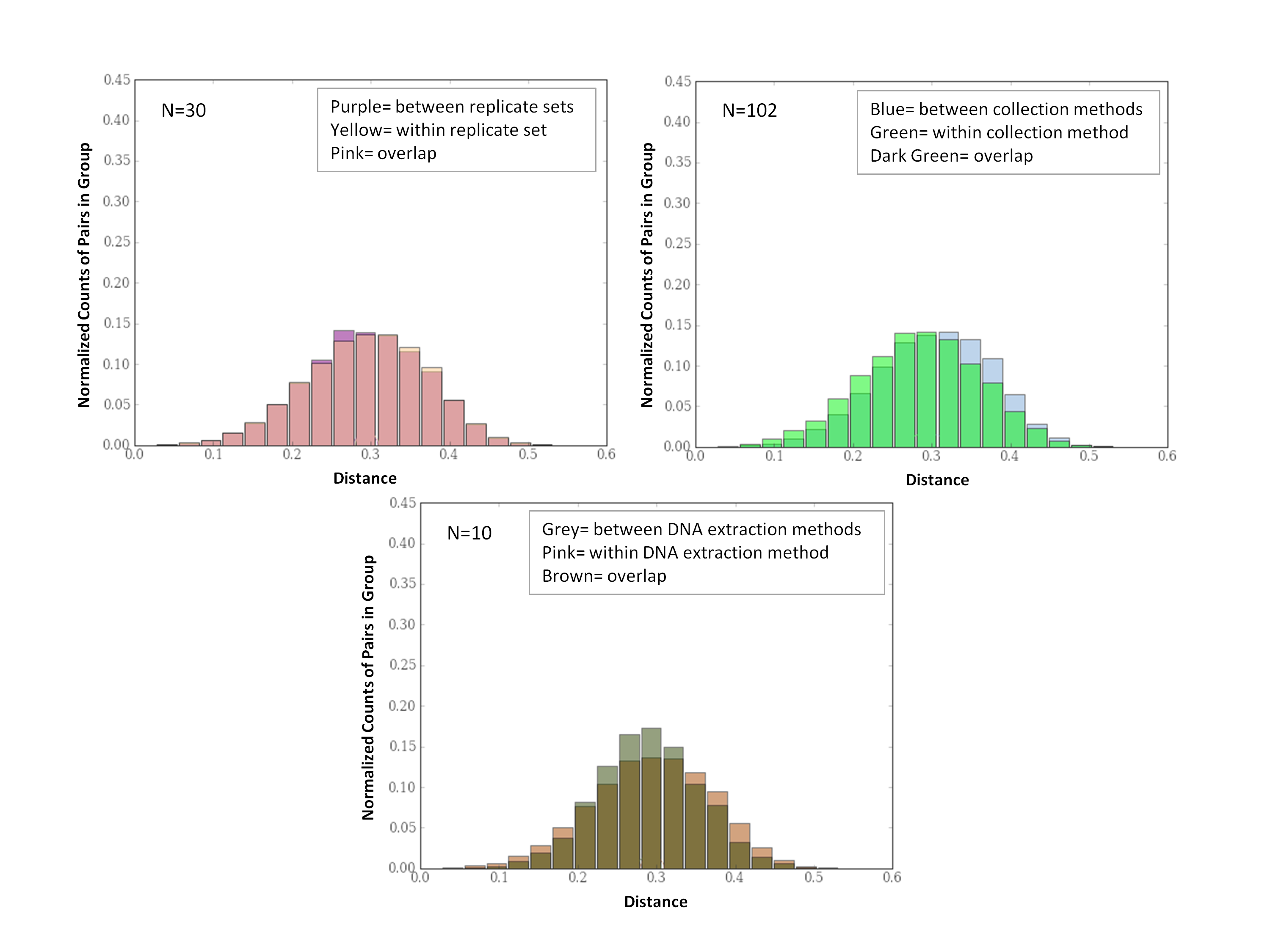

Supplement: Figure S5 — Distribution of Weighted UniFrac Distances Between and Within Each Comparison Group. Weighted UniFrac distance histograms show distribution of distances within sequencing replicate sets similar to the distribution of distances between them. The distribution of distances within sampling collection method was shifted from the distribution of distances between them. The distribution of distances within DNA extraction method was slightly different than the distribution of distances between them. (TIF) [file pone.0088999.s005.tif]

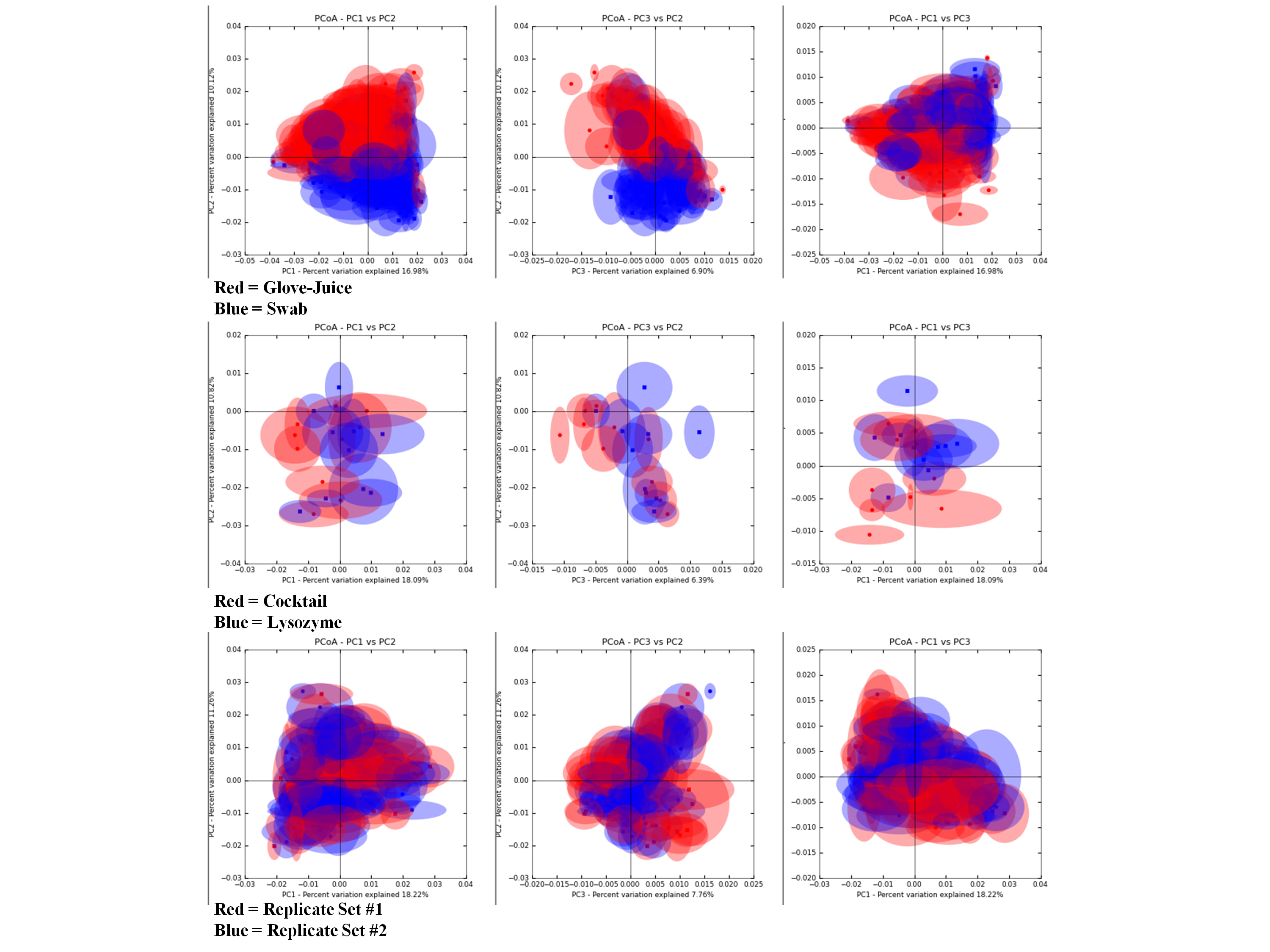

Supplement: Figure S7 — Jackknifed Principal Coordinate Analysis (weighted UniFrac) per Replicate, Sampling Collection Method, and DNA Extraction Method. PCoA performed with jackknife bootstrapping shows considerable overlapping of both sequencing replicate sets as well as DNA extraction methods, but relative clustering by sampling collection method. (TIF) [file pone.0088999.s007.tif]
